# Round the Hospitals

**Published:** 1918-08-10

**Authors:** 


### ROUND THE HOSPITALS.

The banner of Queen Alexandra's Imperial Military Nursing Service was formally dedicated on July 29 at a service held in the chapel of the Queen Alexandra Military Hospital at Millbank. Queen Alexandra was present herself on the occasion, with Princess Victoria, and was received by Lieut.-Gen. Sir Francis Lloyd, Lieut.-Gen. Sir J. H. G. C. Goodwin, Colonel F. R. Buswell, Dame Ethel Becher, and Miss C. Mackay, matron of the Queen Alexandra Military Hospital. The service, conducted by the Archdeacon of London, assisted by the Rev. R. Bartlett and the Rev. J. C. Knapp, was brief but very impressive. The banner has already in its brief history gathered many solemn associations. It will be remembered that at the Commemoration of the First Seven Divisions it was hung among others clothed with glory in the Albert Hall. After the ceremony was over the invalided nurses who were patients in the hospital were cheered by a visit from Queen Alexandra.

Princess Beatrice appeals on behalf of the Central Depôt Surgical Branch of Queen Mary's Needlework Guild for silver ornaments, coin, and even trifling trinkets, which may be sent to her at 2 Cavendish Square, W. 1. This depôt has

**ROUND THE HOSPITALS—*(continued)*.**

sent four and a quarter million articles during the past twelve months to military hospitals at home and abroad. The recent severe fighting has occasioned extra demands to be made, and all who have silver "litter" lying by idle should help the good cause by turning out their treasure-boxes.

The world is echoing with the valiant deeds of our women in France. The award of the Military Medal to sisters and nurses exposed to the horror of the bombing attacks by the enemy on Red Cross hospitals at Etaples have been confidently expected and have been approved by the King. The list of heroic women includes the following: Sister C. E. A. Robinson, A.R.R.C., Q.A.I.M.N.S., who at very great personal risk went in among the ruins of the hospital wing in which many patients were buried and assisted in recovering the patients quite regardless of danger, displaying magnificent courage and resource; Sister N. Galvin, Q.A.I.M.N.S.R., who was on night duty in a ward on which four bombs were dropped; she remained attending to the sick, several of whom were wounded by the bombs, as if nothing had happened, displaying the greatest coolness and devotion to duty; Sister M. M. de Guerin, Q.A.I.M.N.S.R., was on night duty in a ward cut in two by four bombs; she remained on duty in the ward, displaying the greatest coolness and courage in attending to the wounded and helping to rescue the buried; Sister L. A. Wilkinson, Q.A.I.M.N.S.R., who although her ward was demolished during the raid continued while it was still in progress to attend to the wounded; Staff Nurse B. Dascombe, Q.A.I.M.N.S.R., who when her ward was destroyed by a bomb and herself wounded insisted on remaining at her post and attending to the wounded; Matron L. M. M. Toller, R.R.C., Q.A.I.M.N.S., who when the sisters' quarters were wrecked and nurses wounded collected the staff and placed them in comparative safety, and by her fine example undoubtedly saved life; Staff Nurse A. M. McGrath, Q.A.I.M.N.S.R., who when in charge of a ward of serious cases during an air-raid showed a quiet confidence and set a fine example; Sister M. E. Davis, Q.A.I.M.N.S., who when the sisters' quarters were wrecked and bombs were falling showed a fine example in helping to control the situation and attending to the wounded sisters; Staff Nurse S. D. Monroe, who showed coolness, contempt of danger, and invaluable solicitude for patients in a raid which wrecked three of her wards; Staff Nurse K. R. Lowe, T.F.N.S., who when bombs destroyed a large portion of the ward in which she was on night duty continued to carry out her duties with great resourcefulness.

No hospital suffered more that awful night than the one attached to the St. John Ambulance Brigade. Many awards of the Military Medal were made to the devoted sisters in this building. Of Assistant-Matron Chittock it is recorded that she displayed great presence of mind and instilled courage and confidence; of Matron G. E. Todd that she moved freely about the wards during the bombing, encouraging the sisters and patients and displaying great bravery and presence of mind; of Sister Warner that she displayed the utmost coolness and maintained a cheery spirit throughout the raid; of Sister J. Bemrose that she showed disregard of danger and continued to attend the wounded during the heavy bombardment; of Sister McGinnis that she showed great courage, took charge of a ward during the raid, and sustained her patients; of Sister M. H. Ballance that her fortitude and courage were most conspicuous and that she devoted herself entirely to her patients; of Nurse E. Hounlow, A.R.R.C., that when a bomb fell between two of her wards and injured many patients she behaved with the utmost coolness and set a fine example, attending wounded under most trying circumstances; of V.A.D. M. Cavanagh, who was in charge of four wards, two of which were entirely wrecked, that she continued to perform her duty and was very active in removing the wounded to comparative safety.

Mr. H. A. L. Fisher made an eloquent plea for new ideas and original methods in the address which he delivered on August 2 at the opening of the Summer School for Teachers of Young Children, held by the Froebel Society at Westfield College, Hampstead. The founding of nursery schools, which in the new Education Bill is permissive only, ought to bring an immense impetus to the voluntary movements in favour of giving specialised attention to children between the ages of two and five. Mr. Fisher has been very well advised to attempt no centralised plan in regard to these nursery schools. He wants suggestions and offers from people specially interested. He wants more experiments and a far wider knowledge of results than is at present available before making such schools a part of the public elementary school system. That nurses must have their share in the work is universally recognised. And the same "great patience, delicacy, intuition, and intelligence" which Mr. Fisher demands in the teachers must be the passport of the nurse also to success in this branch of work. Most important of all, her mind ought to be inspired by visions of health and gladness, instead of being cramped by melancholy anticipations of sickness and harm.

Miss Carrie Hall is to be congratulated on the fine domain secured to her through the American Red Cross for the convalescence of the United States nurses serving in the war. Colebrook Lodge, near Putney Heath, formerly occupied by Mr. John T. Ryan, of Toronto, is a good modern building, admirably adapted for the purposes of a convalescent home, yet it does not lack the touch of antiquity dear to the heart of the American, for its foundations were laid in Elizabethan or early Jacobean days. The gardens and spacious lawns cover nearly three acres, and it is hard to imagine, while enjoying these pastoral glades, that London is hard by. Miss Hall is herself in direct charge of the establishment, where twenty-five nurses can be accommodated, a number which will

**ROUND THE HOSPITALS**—(*continued*).

be added to when the necessary extension can be carried out. It can hardly be doubted that the directors of similar undertakings in England will find something to imitate and much to admire in this new convalescent home for nurses.

THE Japanese Red Cross Mission now in America will shortly visit England, France, and Italy to study Red Cross methods. It consists of ten members under Prince Tokugawa. Mr. Henry P. Davison, in welcoming the mission at Washington, alluded to the fact that the Red Cross was more fully developed by far in Japan before the war than in any other country, and numbered even then 1,800,000 members. Japanese nurses have long been at work in Paris, and are noted for their delicacy of touch and rapidity in work. Their technique differs in many respects from that of their European *confrères*, but the true spirit of nursing distinguishes them. They work with the heart as well as with the head.

NURSES who are spending their holidays in Devonshire and Cornwall should apply to Mr. E. W. Sexton at the Aquarium, the Hoe, Plymouth, for particulars about collecting red sea-weeds, carrageen, or Irish moss. The stuff is to be found at low water on rocky shores, and when dried and properly treated, makes an excellent gelatine substitute for making jellies for the wounded in Red Cross hospitals. Every new substitute discovered is a step in the right direction, and it may be that carrageen has come to stay long after its immediate use has been abrogated.

A VERDICT of "Wilful murder" was returned against Nurse Thompson on July 30 at the resumed inquest on the body of Kenneth Frederic Cedric Goodman, aged fourteen months, one of the four children who died recently at the Sydenham Infants' Welfare Centre. At inquiries which have been held on the other children serious allegations of ill-treatment were made. Nurse Thompson was the night nurse in charge of the children. Her previous career had been unsatisfactory, the matron of the Oxford Hospital having recently reported to Sister Payne, the superintendent of the Welfare Centre, that the nurse was found on one occasion by the night nurse either drunk or drugged. On another occasion she was found dashing her head against the floor and actually fractured her own skull. Other evidence was given tending to show her unsuitableness for the position she occupied at Sydenham. It is suggested that the heads of the four children who died there were injured by blows from a feeding-bottle which was produced in court.

THE decoration of the Royal Red Cross has been conferred by the King on Assistant Matron Ada Taylor, T.F.N.S.; Assistant Matron Isabel Kemp, Civil N.S.; Sister Elizabeth Macaulay, Civil N.S.; and Matron Myra Goodeve, Civil N.S. The King conferred the Military Medal for courage under fire on Sister Mary Brown, Q.A.I.M.N.S.R. His Majesty admitted the following ladies to be Associates of the Order of the Royal Red Cross:—*Q.A.I.M.N.S.R.*: Sister Georgina Hester, Sister Florence Hughes, Sister Clara Robinson, Sister Amy McDowell, Sister Mercy Huffer, Sister Euphemia Lorraine, Sister Margaret Pierson. *T.F.N.S.*: Sister Charlotte Fitz-Mayor. *Civil Nursing Service*: Matron Ethel Carew-Hodge, Matron Marianne Iffland, Matron Phillimore Ind, Matron Kathleen Irwin, Matron Mabel Johnson, Matron Amy Kaye, Matron Ellen Kidson, Matron Blanche Knapton, Matron Edith Wake, Matron Marie Wheeler, Assistant Matron Isabel Heberden, Sister Ellen Howard, Sister Gertie Inman, Sister Minnie Jones, Mrs. Marian McGlashan. *B.R.C.S.*: Matron Isabel Hunt, Sister Jessie Gunn, Sister Kate Hatton, Sister Kathleen Nixon. *Canadian R.A.N.S.*: Sister Alba Andrew, Sister Irene Brady, Sister Sophie Hoerner.

THE Liverpool Royal Infirmary has decided to accept for a three years' course of training a limited number of V.A.D. nursing members who have served in military hospitals for a period of not less than two consecutive years. They will be admitted under the same condition as paying probationers, but without payment of the usual fees. Suitable regular probationers are now admitted at the age of twenty-one.

WE learn with satisfaction that the salaries of the sisters at the Taunton and Somerset Hospital underwent a substantial increase just two years ago. They were raised to £40 per annum for sisters who had served under two years, and £45 after two years' service. Prior to this increase the sisters had been receiving £30 per annum on appointment, rising to £35. The salaries of the probationers were not at that time increased, but it will be remembered that we recorded the fact in our issue of the 20th ult. that these had recently undergone revision on a generous scale.

NOW that municipalities are establishing maternity homes, the question arises as to the people entitled to admission. This point has been dealt with at Lewisham. The Medical Officer of Health has already received a large number of applications for admission. Some women were disqualified because they did not live in the borough. Of the others sixteen have been found suitable either because of the condition of their homes, or of pecuniary circumstances, and careful inquiry will be instituted in each case. In view of the fact that in many cases an immediate decision will be necessary, it has been decided that applications shall be left in the hands of the Medical Officer of Health, and that the cases accepted for admission shall be duly reported to the sub-committee in charge of the procedure of admission.

☛ For the "Silence of the Nurse," by "Ierne," see p. 410; "The London Hospital Nurses: The Nineteenth and Twentieth Centuries," see p. 415; Matrons' and Sisters' Appointments, see p. 421.

